# Effect of neutrophil to lymphocyte ratio on prognosis of elderly patients with severe sepsis combined with diabetes mellitus

**DOI:** 10.1186/s12877-024-04757-0

**Published:** 2024-02-29

**Authors:** Shan Jin, Jun-bin Yin, Wei Li, Li-li Zang

**Affiliations:** Department of Neurology, Shandong Province 960th Hospital of the People’s Liberation Army, 250031 Jinan, Shandong China

**Keywords:** Severe sepsis, Diabetes mellitus, Elderly patients, Neutrophil-to-lymphocyte ratio (NLR), Short-term prognosis

## Abstract

**Background:**

To investigate the predictive value of neutrophil-to-lymphocyte ratio (NLR) in the short-term prognosis of elderly patients with severe sepsis combined with diabetes mellitus (DM).

**Methods:**

The clinical data of 162 elderly patients with severe sepsis combined with DM from January 2018 to December 2022 were retrospectively collected. These patients were divided into a survival group (*n* = 104) and a death group (*n* = 58) according to 90-day prognosis. The number of neutrophils, lymphocytes, and NLR were compared. The optimal cut-off value for NLR to predict 90-day prognosis in elderly patients with severe sepsis combined with DM was determined using Receiver Operator Characteristic (ROC) curves, and the patients were divided into high and low NLR groups depending on the optimal cut-off value. The Kaplan-Meier method was used to plot the survival curves of the high and low NLR groups. Risk factors for the 90-day death in elderly patients with severe sepsis combined with DM were analyzed by a multivariate cox regression model.

**Results:**

There were no significant differences in gender, age, history of hypertension and hyperlipidemia, intensive care unit (ICU) stay, duration of mechanical ventilation, and oxygenation index between the survival group and death group (*p* > 0.05). However, acute physiological and chronic health evaluation II (APACHE II) scores, and sepsis-related organ failure assessment (SOFA) scores were significantly lower in the survival group compared with the death group (*p* < 0.05). In the survival group, neutrophils counts and NLR were much lower than those in the death group, while lymphocytes counts were much higher (*p* < 0.05). ROC curves showed that the optimal cut-off value for NLR to predict 90-day mortality in elderly patients with severe sepsis combined with DM was 3.482. Patients were divided into high NLR and low NLR groups based on whether NLR was ≥ 3.482. In terms of the log-rank test results, patients in the low NLR group had a significantly higher 90-day survival rate than those in the high NLR group (Logrank χ^2^ = 8.635, *p* = 0.003). The multivariate cox regression model showed that the length of ICU stay longer than 15 days and NLR ≥ 3.482 were independent risk factors for 90-day prognosis in elderly patients with severe sepsis combined with DM.

**Conclusion:**

NLR ≥ 3.482 can be used to predict whether poor prognosis occurs in the short term after illness in elderly patients with severe sepsis combined with DM, and has good assessment value.

## Background

Sepsis is a condition caused by a disturbance in the internal environment of the body in reaction to severe infection. In severe cases, the disease can even develop into multiple organ dysfunction syndrome (MODS). Septic patients require comprehensive treatment measures, and lack of early intervention and treatment can lead to death [1]. Each year, millions of people are treated for sepsis [2] and died as a result. A large meta-analysis in 2020 showed that the mortality rate of septic patients exceeded 20% [3]. However, the pathogenesis of sepsis is not yet fully understood. Existing studies have found that systemic inflammatory reaction syndrome (SIRS) can manifest itself early in sepsis, suggesting that sepsis is fundamentally an immune dysfunctional process [4]. When a pathogen invades the organism, the immune system is activated but unable to clear the pathogen. If the immune system is overactivated, a large number of pro-inflammatory mediators will be released to trigger an inflammatory storm [5]. Therefore, assessing the immune system in septic patients will help physicians to take the most active and effective treatment. Neutrophils and lymphocytes are the most important organismal immune cells involved in the whole process of sepsis [6]. The neutrophil-to-lymphocyte ratio (NLR) has been revealed to indicate the prognostic status of septic patients [7].

However, NLR is mostly used to predict the prognosis of all septic patients. As for elderly diabetic patients with high risk of sepsis, the prognostic value of NLR has not been clearly explored. Advanced age or/and diabetes are poor prognostic factors for sepsis, with the elderly accounting for more than 60% of cases [8]. Moreover, the atypical symptoms in elderly patients may delay diagnosis [9, 10]. Diabetes, more seriously, can aggravate sepsis [11]. Therefore, finding prognostic indicators for the detection of severe sepsis in elderly patients with diabetes mellitus would be crucial for their clinical management. In this study, we investigated the predictive value of NLR in the prognosis of 162 elderly patients with severe sepsis combined with diabetes mellitus from our hospital by retrospectively analyzing their clinical data.

## Subjects and methods

### Study subjects

A retrospective collection of 162 elderly patients with severe sepsis combined with diabetes mellitus diagnosed and treated in our hospital from January 2018 to December 2022 was performed. These patients were divided into a survival group (*n* = 104) and a death group (*n* = 58) according to the 90-day prognosis. Inclusion criteria: patients with (1) age of ≥ 60 years; (2) a history of diabetes mellitus; and (3) a diagnosis of severe sepsis. Exclusion criteria: (1) those who died within 24 h after admission; (2) those with a leukocyte count < 0.5 × 10^9^/L; (3) those with severe underlying diseases; (4) those with serious diseases of organs such as the heart and kidney; (5) those with autoimmune diseases, hemopathy, immunodeficiency who received chemotherapy and treatment with immunosuppressants or monoclonal antibodies. This study was approved by the ethics committee of Shandong Province 960th Hospital of the People’s Liberation Army (2023-058). All patients were fully informed and signed the consent form for donation of biological sample and information at the time of admission.

### Data collection

Basic information: gender, age, comorbidity (hypertension, hyperlipidemia), acute physiological and chronic health evaluation II (APACHE II) score, sepsis-related organ failure assessment (SOFA) score, intensive care unit (ICU) stay, duration of mechanical ventilation, and oxygenation index were collected for all included patients.

Neutrophils, lymphocytes and NLR: Peripheral venous blood was collected on the day the patients were admitted to the hospital, and routine blood tests were performed using a WD.5000 Auto Hematology Analyzer (Jilin WEIER Medical Equipment Co., ltd.). Neutrophil counts and lymphocyte counts were recorded. NLR = neutrophil count/lymphocyte count.

Basic vital signs: The basic vital signs including respiratory rate, heart rate, and mean arterial pressure (MAP) were collected on the day of admission, day 3, day 5, and day 7 for both groups.

### Statistical analysis

The statistical software GraphPad® 9.5 was used. Continuous variables were expressed as mean ± standard deviation (SD), and the t-test was performed to compare the differences between two groups; categorical variables were expressed as n (%), and the chi-square test was used to compare the differences between two groups. Receiver Operator Characteristic (ROC) curves were plotted to determine the optimal cut-off value for NLR to predict 90-day mortality in elderly patients with severe sepsis in combination with diabetes. The obtained cut-off value was applied to grouping (high NLR and low NLR groups). The Kaplan-Meier method was used to plot survival curves. Hypothesis testing was performed to examine the survival curves of the high NLR and low NLR groups using the log-rank test. A multivariate cox regression model was contracted to analyze the risk factors for the 90-day mortality in elderly patients with severe sepsis combined with diabetes. *P* < 0.05 was considered to be statistically significantly different. In this study, we set the level of significance (two-sided) as ɑ = 0.05 and effect size d = 0.5. The power value was calculated using G*Power 3.1 software as 0.858.

## Results

### Basic information of patients in both groups

**Fig. 1:**
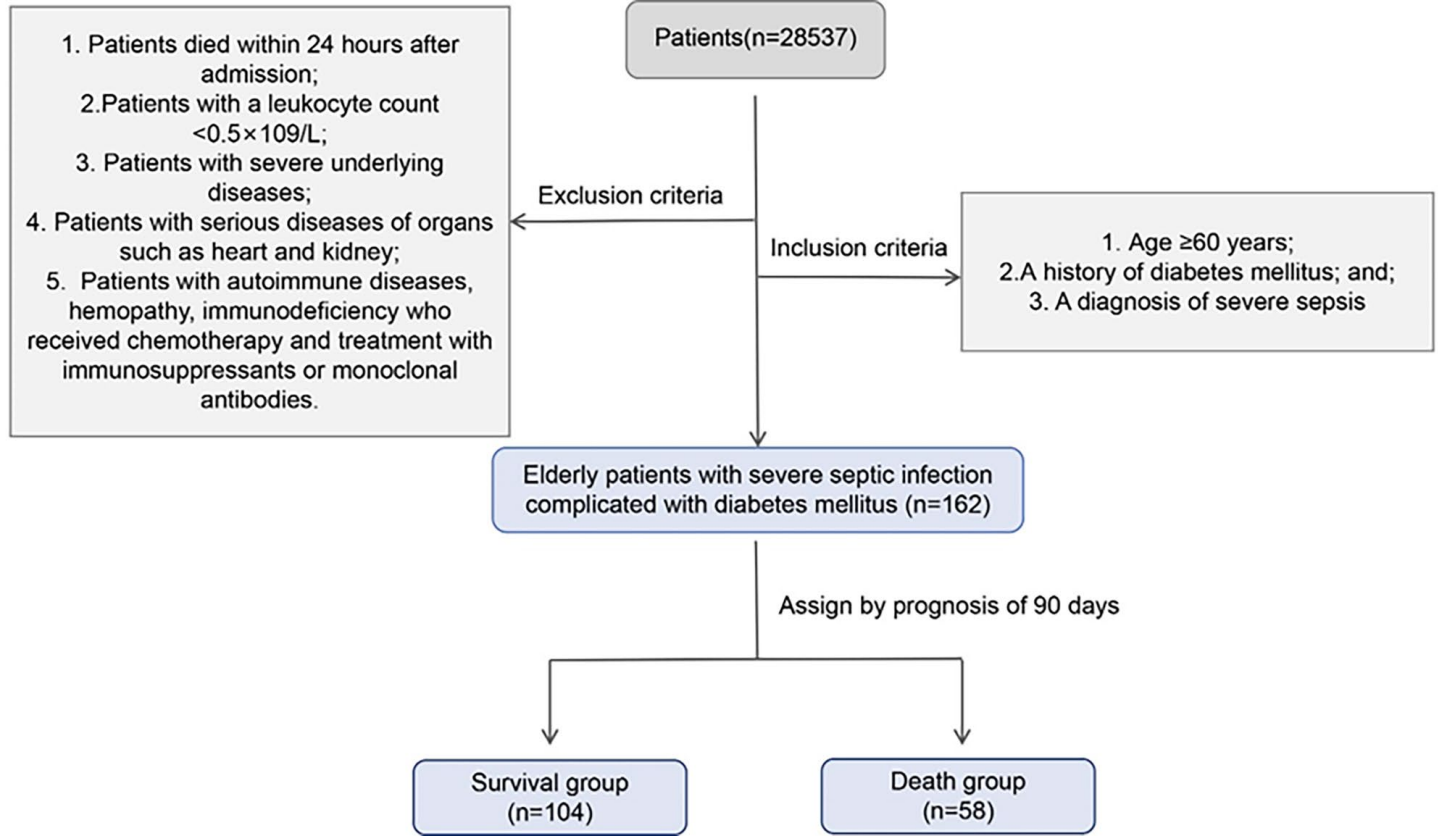
Flow chart of the study

A total of 162 elderly patients with severe sepsis and comorbid diabetes mellitus who met the inclusion criteria were collected at our hospital from January 2018 to December 2022 and were divided into a survival group (*n* = 104) and a death group (*n* = 58) depending on the 90-day prognosis of the patients, Flow chart of grouping is shown in Fig. 1. The collection results are shown in Table 1. The gender (*p* = 0.997), age (*p* = 0.167), history of hypertension (*p* = 0.302), the history of hyperlipidemia (*p* = 0.55), ICU stay (*p* = 0.156), duration of mechanical ventilation (*p* = 0.346) and oxygenation index (*p* = 0.957) were not significantly different in the two groups (*p* > 0.05). However, APACHE II score (37.67 ± 4.09 vs. 39.55 ± 5.17; *p* = 0.012) and SOFA score (8.51 ± 2.91 vs. 9.66 ± 2.31; *p* = 0.011) were much lower in the survival group compared with the death group.

**Table 1:** Basic information of patients in both groups

	Survival group (*n* = 104)	Death group (*n* = 58)	X^2^/t	p-value
Age (years)	70.81 ± 4.00	71.74 ± 4.28	-1.390	0.167
Gender (%)			0.000	0.997
men	61(58.7)	34(58.6)		
women	43(41.3)	24(41.4)		
Hypertension (%)			1.067	0.302
no	50(48.1)	23(39.7)		
yes	54(51.9)	35(60.3)		
Hyperlipidemia (%)			3.668	0.055
no	68(65.4)	29(50.0)		
yes	36(34.6)	29(50.0)		
ICU stay (days)	7.24 ± 2.67	7.90 ± 3.04	-1.425	0.156
Mechanical ventilation (days)	3.49 ± 0.97	3.34 ± 0.89	0.946	0.346
Oxygenation index	239.17 ± 17.83	239 ± 22.71	0.054	0.957
APACHEII score	37.67 ± 4.09	39.55 ± 5.17	-2.545	0.012
SOFA score	8.51 ± 2.91	9.66 ± 2.31	-2.575	0.011

Values are expressed as mean ± SD or number (%). ICU: intensive care unit; APACHE II: acute physiological and chronic health evaluation II; SOFA: sepsis-related organ failure assessment

### Comparison of neutrophil count, lymphocyte count and neutrophil to lymphocyte ratio (NLR) in the two groups

**Fig. 2:**
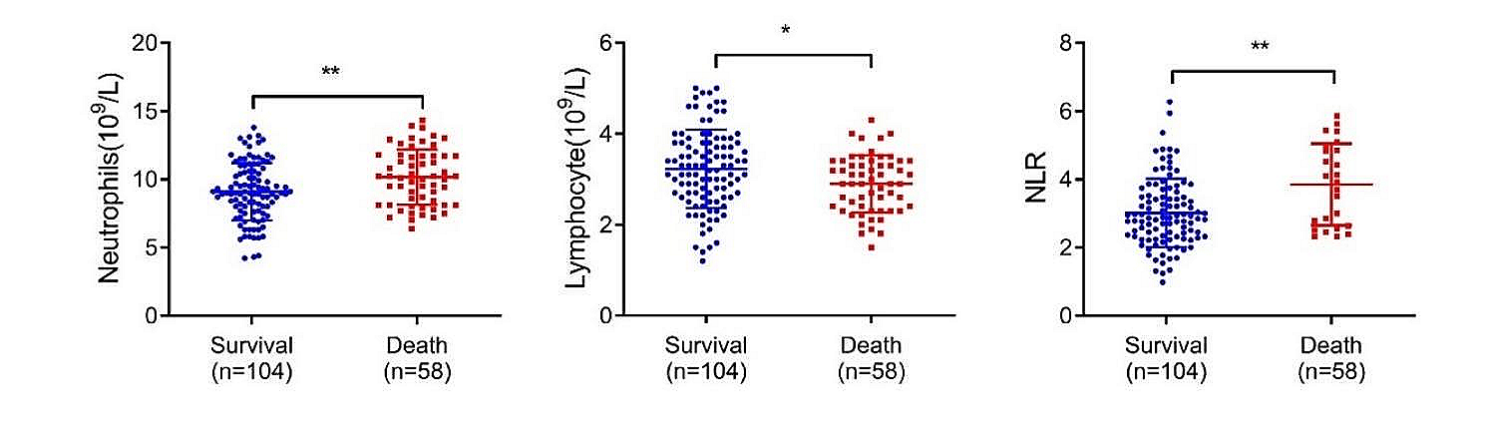
Neutrophil count, lymphocyte count and NLR in two groups. NLR: neutrophil to lymphocyte ratio. *p < 0.05; **p < 0.001

The results are shown in Table 2; Fig. 2. The survival group showed much lower neutrophil counts (9.09 ± 2.11 vs. 10.15 ± 2.04; *p* = 0.002) and NLR (3.01 ± 1.00 vs. 3.68 ± 1.16; *p* < 0.001) than the death group, while much higher lymphocyte counts (3.23 ± 0.86 vs. 2.90 ± 0.63, *p* = 0.011).

**Table 2:** Comparison of neutrophil count, lymphocyte count and NLR in the two groups

Groups	n	Neutrophil count (10^9^/L)	Lymphocyte count (10^9^/L)	NLR
Survival group	104	9.09 ± 2.11	3.23 ± 0.86	3.01 ± 1.00
Death group	58	10.15 ± 2.04	2.90 ± 0.63	3.68 ± 1.16
*t*		-3.107	2.563	-3.893
*p-value*		0.002	0.011	< 0.001

Values are expressed as mean ± SD. NLR: neutrophil to lymphocyte ratio

### Predictive value of NLR for 90-day prognosis in elderly patients with sepsis combined with diabetes mellitus

**Fig. 3:**
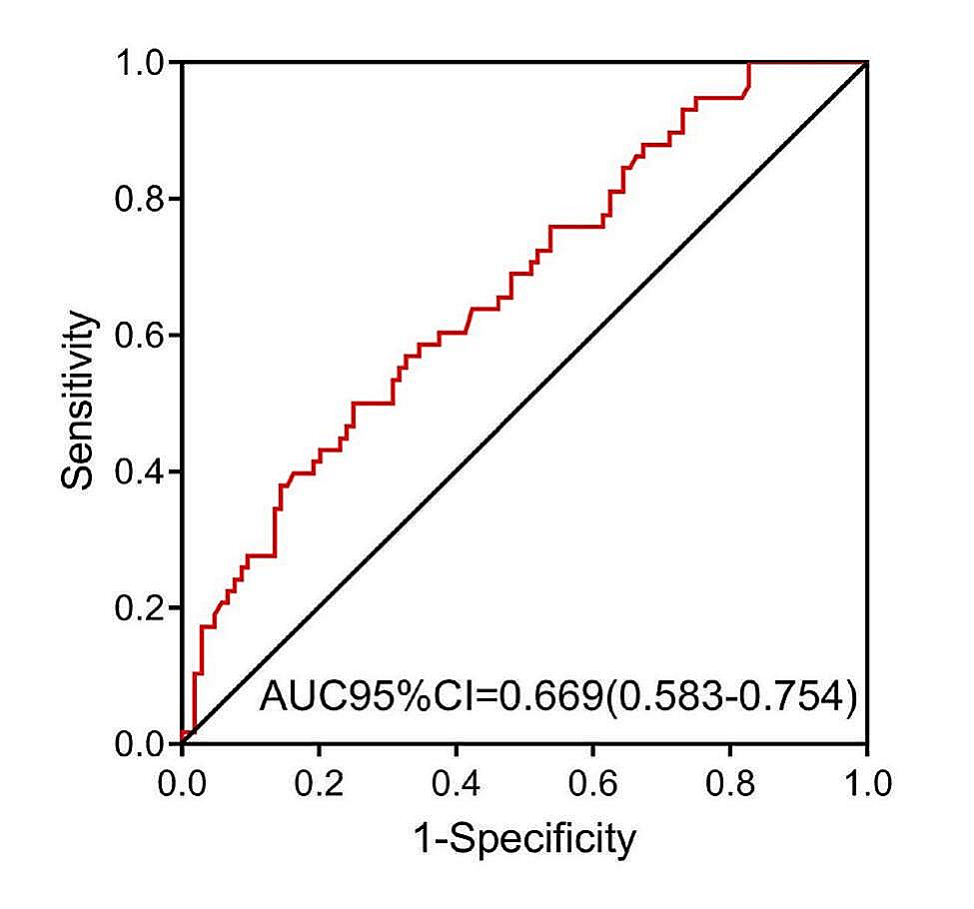
Predictive value of NLR on 90-day prognosis of sepsis in elderly patients with sepsis combined with diabetes mellitus

Because NLR was significantly different in the survival and death groups, we plotted ROC curves to calculate the optimal cutoff value for NLR to predict 90-day mortality in elderly patients with severe sepsis combined with diabetes mellitus. As a result, we obtained that the optimal cutoff value was 3.482, and that the sensitivity, specificity, area under curve (AUC), positive predictive value and negative predictive value were 50.0%, 75.0%, 0.669 (*p* < 0.001), 52.7% and 72.9%, respectively (Table 3; Fig. 3).

**Table 3:** Predictive value of NLR on 90-day prognosis of sepsis in elderly patients with sepsis combined with diabetes mellitus

	AUC	95%CI	p-value	Sensitivity (%)	Specificity (%)	Positive predictive value (%)	Negative predictive value (%)	Cutoff value
NLR	0.669	0.583–0.754	< 0.001	50.0	75.0	52.7	72.9	3.482

NLR: neutrophil to lymphocyte ratio; AUC: area under curve; CI: confidence interval;

### Differences in basic vital signs between the high NLR and low NLR groups

**Fig. 4:**
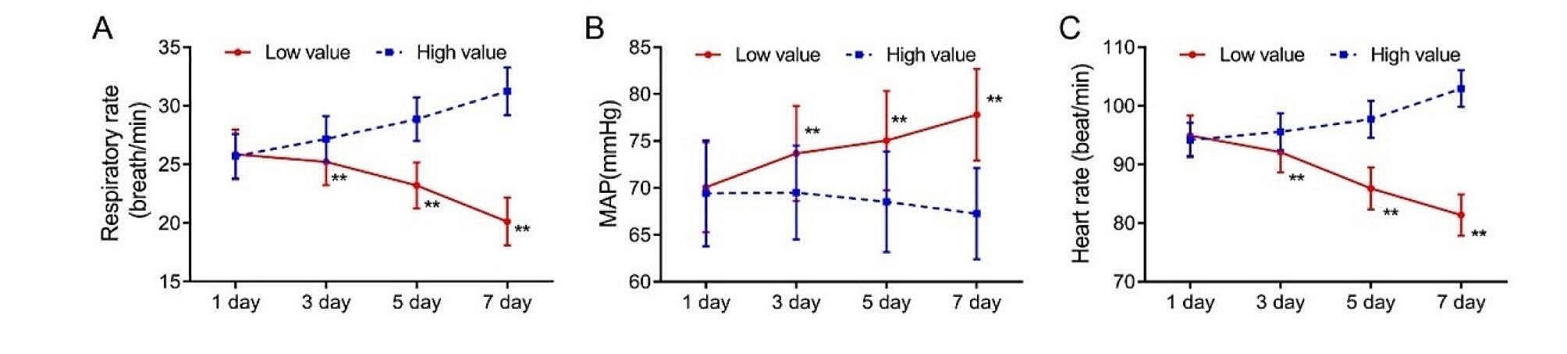
Differences in basic vital signs between the high NLR and low NLR groups MAP: mean arterial pressure; **p < 0.01, vs. high NLR group

As shown in Table 4, the optimal cut-off value for NLR to predict 90-day mortality in patients was 3.482, and the patients were divided into high NLR and low NLR groups based on the optimal cut-off value. On the first day of admission, there was no statistically significant difference in respiratory rate (*p* = 0.658), MAP (*p* = 0.433) and heart rate (*p* = 0.189) between the two groups. On days 3, 5, and 7, compared with the first day, heart rate (day 3, 5, 7, *p* < 0.001) and respiratory rate (day 3, *p* = 0.026; day 5 and day 7, *p* < 0.001) decreased and MAP (day 3, 5, 7, *p* < 0.001) increased in the low NLR group with statistically significant differences (*p* < 0.05), while heart rate (day 3, *p* = 0.017; day 5 and day 7, *p* < 0.001) and respiratory rate (day 3, 5, 7, *p* < 0.001) increased obviously in the high NLR group. However, the differences in MAP in the high NLR group on days 3 (*p* = 0.999), 5 (*p* = 0.683) and 7 (*p* = 0.067) were not statistically significant when compared with day 1. The differences in respiratory rate, MAP, and heart rate between the two groups were statistically significant (*p* < 0.001) on days 3, 5, and 7 (Fig. 4).

**Table 4:** Differences in basic vital signs between the high NLR and low NLR groups

Variables	Groups	Day 1	Day 3	Day 5	Day 7
Respiratory rate (breath/min)	Low NLR group (*n* = 107)	25.86 ± 2.12	25.23 ± 1.99*	23.21 ± 1.97**	20.13 ± 2.04**
	High NLR group (*n* = 55)	25.71 ± 1.90	27.18 ± 1.96**	28.85 ± 1.85**	31.24 ± 2.04**
	*t*	0.444	-5.921	-17.628	-32.805
	*p-value*	0.658	< 0.001	< 0.001	< 0.001
MAP (mmHg)	Low NLR group (*n* = 107)	70.08 ± 4.80	73.67 ± 5.05**	75.06 ± 5.29**	77.81 ± 4.89**
	High NLR group (*n* = 55)	69.42 ± 5.66	69.51 ± 5.01	68.53 ± 5.35	67.25 ± 4.87
	*t*	0.787	4.982	7.406	13.040
	*p-value*	0.433	< 0.001	< 0.001	< 0.001
Heart rate (beat/min)	Low NLR group (*n* = 107)	94.90 ± 3.45	92.11 ± 3.43**	85.90 ± 3.62**	81.37 ± 3.51**
	High NLR group (*n* = 55)	94.18 ± 2.89	95.58 ± 3.15*	97.71 ± 3.14**	102.96 ± 3.10**
	*t*	1.318	-6.256	-20.556	-38.509
	*p-value*	0.189	< 0.001	< 0.001	< 0.001

Values are expressed as mean ± SD. MAP: mean arterial pressure; *p < 0.05, **p < 0.01, vs. Day 1

### Kaplan-Meier curves of patients in the high NLR and low NLR groups

**Fig. 5:**
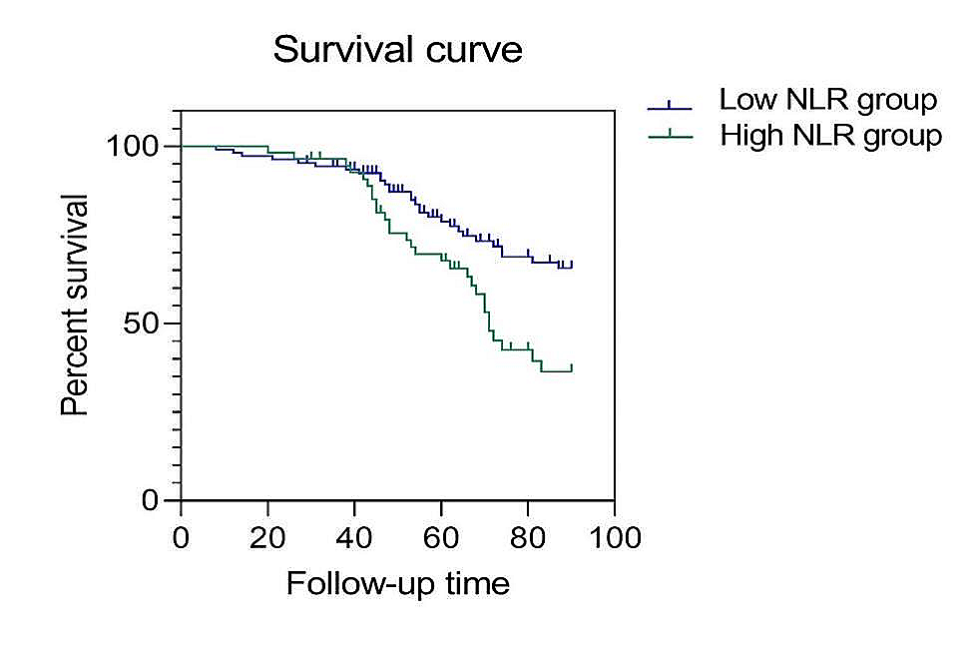
Kaplan-Meier curves of the high NLR group and low NLR group

The overall survival of patients in the high NLR group was 69.5 months, with a 90-day survival rate of 36.5%; the overall survival of patients in the low NLR group was 77.5 months, with a 90-day survival rate of 65.6%. The log-rank test results showed that the low NLR group exhibited a much higher 90-day survival rate than that of the high NLR group, and the difference was statistically significant (Logrank χ^2^ = 8.635, *p* = 0.003) (Fig. 5; Table 5).

**Table 5:** Survival analysis results of the high NLR group and low NLR group

Groups	Total	Event count	OS	95%CI	Logrank X^2^	p-value
Low NLR group	107	29	77.5	73.341–81.680	8.635	0.003
High NLR group	55	29	69.5	63.962–75.038		

OS: overall survival; CI: confidence interval: NLR: neutrophil to lymphocyte ratio

### Risk factors for the 90-day death in elderly patients with severe sepsis in combination with diabetes mellitus

Further, we used COX regression analysis to measure the impact of clinical factors on the risk of death. Hazard ratio (HR) values were used to express the ratio of the risk of death. The NLR grouping and the underlying clinical factors of the patients were included in the Cox univariate analysis. It was discovered that the APACHE II score, SOFA score, ICU stay, and NLR grouping were correlated with the 90-day death in elderly patients with severe sepsis in combination with diabetes mellitus. Further Cox multifactor analysis yielded that NLR ≥ 3.482 (HR = 2.001, 95% confidence interval [CI]: 1.183–3.384, *p* = 0.010) and ICU stay ≥ 15 d (HR = 1.899, 95% CI: 1.08–3.34, *p* = 0.026) could be used as independent risk factors for 90-day mortality in those patients (Table 6).

**Table 6:** Risk factors for the 90-day death in elderly patients with severe sepsis in combination with diabetes mellitus

	Single-factor		Multi-factor	
	HR(95%CI)	p-value	HR(95%CI)	p-value
Age	1.034(0.969–1.103)	0.313		
Gender	1.039(0.616–1.752)	0.887		
ICU stay	1.045(0.960–1.139)	0.307		
Hospital saty	2.157(1.246–3.733)	0.006	1.899 (1.08–3.34)	0.026
Hypertension	1.224(0.723–2.072)	0.452		
Hyperlipidemia	1.632(0.975–2.731)	0.062		
Mechanical ventilation	0.878(0.664–1.162)	0.363		
Oxygenation index	0.997(0.984–1.011)	0.698		
APACHEII score	1.067(1.006–1.131)	0.030	1.052(0.991–1.117)	0.098
SOFA score	1.143(1.040–1.255)	0.005	1.083(0.980–1.197)	0.118
NLR grouping	2.124(1.267–3.560)	0.004	2.001(1.183–3.384)	0.010

ICU: intensive care unit; APACHE II: acute physiological and chronic health evaluation II; SOFA: sepsis-related organ failure assessment; NLR: neutrophil to lymphocyte ratio; HR: hazard ratio; CI: confidence interval

## Discussion

Severe sepsis is a severe infectious state that has a negative impact on patient survival, while diabetic patients have a significantly higher propensity to develop infections than the general population. Therefore, early assessment of the disease progression will influence the ultimate level of prognosis [12, 13]. In this study, we retrospectively analyzed the clinical data of 162 elderly patients with severe sepsis in combination with diabetes mellitus, and the patients were divided into survival and death groups based on their 90-day prognosis. Comprehensive analysis revealed that the APACHE II and SOFA scores of patients in the survival group were significantly lower than those in the death group. Studies have shown that the APACHE II score and SOFA score are independent risk factors for patients with sepsis [14]. A meta-analysis confirmed that septic patients who developed acute respiratory distress syndrome (ARDS) had significantly higher APACHE-II and SOFA scores than non-ARDS patients [15]. This is consistent with our findings, which suggest that APACHE-II and SOFA scores are important prognostic indicators of death in critically ill patients.

Furthermore, we found that patients in the survival group had significantly lower NLR than those in the death group. Patients with high NLR responded more poorly to treatment. And it was challenging to lower heart rate and respiratory rate, as well as elevate MAP during treatment to maintain the basic vital signs. The difference in NLR between the death and survival groups was mainly attributed to the higher neutrophil and lower lymphocyte levels in the death group. This phenomenon was also discovered in earlier investigations of all septic patients [7, 16]. Our results suggest that NLR is of prognostic significance in elderly patients with severe sepsis in combination with diabetes mellitus. A rise in neutrophil count is often accompanied by a fall in lymphocyte count [17]. Patients may experience a mismatch between elevated neutrophil numbers and the severity of infection in some specific circumstances, such as when they are combined with cachexia or immunosuppression [18]. In contrast, NLR reflects the online dynamic relationship between intrinsic (neutrophils) and adaptive cellular immune responses (lymphocytes) in disease and various pathologic states [19]. A retrospective analysis by Sun et al. demonstrated that NLR is essential for the early detection of sepsis induced by diabetic foot ulcers and is an independent predictive marker of their prognosis [20]. Hence, NLR is a more comprehensive and reliable predictor than neutrophil count alone.

Based on the above results, we further analyzed NLR. ROC curve analysis showed that the AUC of NLR for predicting 90-day mortality in elderly patients with severe sepsis in combination with diabetes mellitus was 0.669. Although the AUC value was not high, it was greater than 0.5 and significantly different, which suggests that NLR has some value in predicting 90-day mortality in patients. Subsequently, we found the most appropriate cutoff value of 3.482, at which point the specificity (75%) was higher than the sensitivity (50%). These results show NLR predicts a low misdiagnosis rate for patients who experience death. Further, patients were divided into high and low NLR groups based on whether NLR was ≥ 3.482. Multivariate cox regression analysis results showed that NLR ≥ 3.482 was an independent risk factor for 90-day prognosis in elderly patients with severe sepsis in combination with diabetes mellitus. However, APACHE-II and SOFA scores were not significantly associated with the occurrence of death. As for other studies, NLR level in normal population is found to be generally 1.6–1.7 [21, 22]. Our study uncovered that NLR ≥ 3.482 indicated poor prognosis in elderly patients with sepsis in combination with diabetes mellitus. The NLR is greater according to the results of previous studies on the predictive value of NLR in sepsis, with half of the studies suggesting that NLR could reach 10 [23]. We speculate that this may be due to the fact that the subjects in this study were elderly diabetic patients with worse basal immune levels. NLR levels considered acceptable in the general population are already predictive of poor prognosis in the high-risk population. It is generally accepted that as people age, their immune systems weaken, and they become more vulnerable to infections. Unfortunately, the effects of age on neutrophils and lymphocytes are not yet fully understood. Moreover, the immune cell function is similarly altered with age [24, 25]. The relationship between NLR and diabetic complications has been demonstrated. To be more precise, a high NLR (or neutrophil count) in hyperglycemic conditions is correlated with the thrombosis [26] and susceptibility to sepsis [27] in mouse models. An association between high NLR and complications in diabetic patients was also discovered in epidemiological statistics [28]. All of these results imply that more stringent observed indicators should be used for the elderly diabetic patients, rather than those used for the general population.

There are some limitations to this study. Firstly, this was a a single-center study, and no external validation of the results was done. In the future, external data should be used for validation. Secondly, this study only investigated the short-term mortality of patients. Previous studies have reported the impact of sepsis on the long-term prognosis of patients. Therefore, patients should receive longer-term follow-up. Finally, the underlying disease causing sepsis may differ between patients. It is best to prevent infections from different sources in various diseases.

## Conclusion

NLR ≥ 3.482 has good assessment value and should be encouraged in clinical practice since it can effectively predict poor short-term prognosis in elderly patients with severe sepsis in combination with diabetes mellitus.

## Data Availability

Data used in this study are available from the corresponding author upon reasonable request.
